# Editorial: Otitis Media Genomics and the Middle Ear Microbiome

**DOI:** 10.3389/fgene.2021.763688

**Published:** 2021-10-12

**Authors:** Regie Lyn P. Santos-Cortez, Garth D. Ehrlich, Allen F. Ryan

**Affiliations:** ^1^ Department of Otolaryngology-Head and Neck Surgery, School of Medicine, University of Colorado Anschutz Medical Campus, Aurora, CO, United States; ^2^ Center for Children’s Surgery, Children’s Hospital Colorado, Aurora, CO, United States; ^3^ Institute for Molecular Medicine and Infectious Disease, Drexel University College of Medicine, Philadelphia, PA, United States; ^4^ Departments of Otolaryngology-Head and Neck Surgery, and Microbiology and Immunology, Drexel University College of Medicine, Philadelphia, PA, United States; ^5^ Division of Otolaryngology, Department of Surgery, University of California San Diego School of Medicine, La Jolla, CA, United States; ^6^ Veterans Affairs Medical Center, La Jolla, CA, United States

**Keywords:** gene, microbiome, middle ear, nasopharynx, otitis media (OM), sequencing

## Otitis Media as a Clinical Disease and a Public Health Issue

Otitis media (OM), defined as inflammation of the middle ear (ME), usually occurs after ME infection and may cause hearing loss (HL) at any age, but most frequently in early childhood when development of speech and cognition occurs (Davis and Hind, 1999; Chonmaitree et al., 2016; Singer et al., 2018). Globally in 2019, 64% of children 1–5 years have HL due to OM (GBD, 2021). Antibiotics are prescribed for 67–98% of children with OM (Hersh et al., 2016; Frost et al., 2020), which contributes to antibiotic resistance and drug-specific adverse effects, even if administered appropriately (Fleming-Dutra et al., 2016; DeMuri et al., 2017; Islam et al., 2020). 4–10% of children require surgery due to recurrent acute (RA) OM or chronic OM with effusion (COME) (Beyea et al., 1999; Bhattacharyya and Shay, 2020). RAOM is diagnosed in children with ≥3 OM episodes in 6 months or ≥4 in 12 months, and COME if ME effusion behind the intact eardrum persists for >2 months (Rosenfeld et al., 2016). Combined, these OM types and surgery are associated with 4–11× risk of permanent HL (Rach et al., 1988; Beyea et al., 1999). RAOM/COME cause defects in language ability, auditory perception, sound localization and auditory processing in 5–11% of school-age children, which impedes academic progress (Morrongiello, 1989; Zargi and Boltezar, 1992; Moore, 2007; Hind et al., 2011; Skarzynski et al., 2015; Graydon et al., 2017; Cordeiro et al., 2018; Leach et al., 2020). In 10–24% of chronic OM cases a highly destructive, cyst-like ME lesion, cholesteatoma, can later grow and erode functionally critical bony and neural structures (Schmidt Rosito et al., 2017). Complications of OM/cholesteatoma besides HL include balance disorders, facial nerve paralysis, soft tissue abscess, or intracranial infection (O’Connor et al., 2009). Cholesteatoma is treated surgically, but often recurs (Kuo et al., 2012; Nardone et al., 2012). Novel strategies for prevention and early treatment of OM are needed to decrease the health burden worldwide due to OM and HL. Unfortunately, OM and its comorbidities remain severely understudied compared to other common diseases.

OM phenotypes (acute OM, RAOM, COME, chronic OM, cholesteatoma) are not distinct entities but fall within a spectrum. However, the mechanisms by which common acute OM progresses in severity despite treatment are poorly understood. Elucidation of molecular mechanisms of genetic susceptibility in humans, and of host-microbiota interactions are key to understanding OM progression and improving current protocols for prevention, diagnosis and treatment (Marsh et al., 2020; Santos-Cortez et al., 2020; Thornton et al., 2020).

## What This Research Topic Contributes to Scientific Knowledge on Otitis Media

Three reviews in this research topic (Mittal et al.; Geng et al.; Giese et al.) provide comprehensive overviews of the current knowledge on the genomics of host-microbial interactions within the context of OM. They enumerate the genes that have been identified in humans and animal models as predisposing to OM, as well as their effects on the nasopharyngeal and ME microbiotas. Additionally, Lappan et al. summarized what is known about two bacterial species *Alloiococcus otitidis* and *Turicella otitidis* that are identified by sequencing in the ME and/or external ear. They suggested studies to delineate the role of these two bacteria in the ME, including microbiota, transcriptomic and animal model studies to aid understanding metabolic functions, strain heterogeneity, inflammatory effects and changes in OM severity due to *A. otitidis* or *T. otitidis* alone, or in concert with other otopathogens.

### The Otitis Media-Related Microbiotas of the Pediatric Middle Ear and Nasopharynx (NP)

Three microbiota studies were performed using ME and NP samples from children with OM. Brugger et al. showed that the NP microbiota increased in richness but decreased in evenness with age, suggesting a personalized microbiota becoming more similar to adults with time; e.g., increased relative abundance of *Staphylococcus* and *Corynebacterium.* Xu et al. determined that microbiota from nasal washes or ME fluid at onset of acute OM were similar, but lower in biodiversity than the healthy state in the same children 3 weeks prior. These findings highlight the importance of temporal specificity when collecting paired NP and ME samples for OM studies. On a different note, Kolbe et al. found that in children with COME, the ME had less microbial biodiversity if the child had lower respiratory tract (LRT) disease i.e., asthma, bronchiolitis. Children with both COME and LRT disease also had greater relative abundances for *Haemophilus, Staphylococcus* and *Moraxella* but less *Turicella* or *Alloiococcus* in ME fluid. Altogether these ME and NP microbiota findings will be useful as guide in the design and interpretation of future microbial profiling and metagenomic studies.

### The Middle Ear Transcriptome in Health and Otitis Media

Among five studies on ME gene expression, two were performed on human samples while three studies included rodent ME tissues. Microarray profiling of leukocytes from ME fluid of children with COME revealed enriched hypoxia signaling pathways and increased VEGF (compared to blood or plasma) that decreased with age (Bhutta et al.). This was concurrent with upregulation of inflammatory networks and increased myeloid cell signature and neutrophil counts in mucoid ME effusions; in contrast, lymphocytes and eosinophils were higher in serous ME fluid. Baschal et al. performed bulk mRNA-sequencing on human cholesteatoma and ME mucosal tissues from chronic OM patients, compared to published datasets from sinus and LRT. Their main findings include 1,806 differentially expressed genes (DEGs) and 68 enriched pathways in cholesteatoma compared to ME mucosa, as well as DEGs (including novel genes *CR1* and *SAA1*) and pathways that overlap among the ME, upper and lower airways.

Ryan et al. performed the first single-cell RNA-sequencing study of ME tissue, using wildtype, non-infected mice. They extensively described transcriptomic profiles across 22 ME cell types, including genes involved in innate immunity and basic cellular pathways related to infection responses. Zhao et al. studied the endoplasmic reticulum (ER) stress pathway, characterizing histologic, hearing, multi-gene expression and oxidative stress responses in inflamed mouse MEs with or without an ER stress inhibitor. The ME inflammatory responses were reduced by administration of ER stress inhibitor, suggesting ER stress pathways as a potential target for treatment of OM. Similarly, Yadav et al. demonstrated in rat ME that Asian sand dust, in concert with pneumococcal infection, promoted bacterial colonization, biofilm growth, cell apoptosis, ROS production, pro-inflammatory responses, and differential expression of host genes in multiple pathways such as immune defence, cell differentiation and neurogenesis. Expression of microbial genes involved with competence, biofilm and toxin production was also increased. These transcriptome studies serve as seminal references for future identification of novel genes, pathways and responses to various agents contributing to OM.

### Genetic or Environmental Mouse Models and the Otitis Media Phenotype

Three mouse models were reported within this topic. Double-mutant *Id1-Id3* mice had hearing loss, and ME fluid depending on genotype (Zheng et al.). Histologic analysis confirmed ME polymorphonuclear cell infiltration, fibrosis and mucosal thickening, likely due to immune effects of *Id* gene knockout (KO). In a study of *Fbxo11* variants, the *Jeff* mouse which has an *Fbxo11* missense variant developed chronic OM and HL while the KO-mouse only had a milder craniofacial defect and ME mucosal thickening but no ME fluid or auditory phenotype (Kubinyecz et al.). Profile differences in cytokine levels and cell populations for innate or adaptive immunity were also stronger in the *Jeff* mouse than the KO (Vikhe et al.). These disparate phenotypes suggest a gain-of-function effect of the *Jeff* missense mutation. In summary, these mouse models demonstrate downstream effects of changes in the immune and TGF-beta pathways that aid understanding of the OM phenotype.

## Future Perspectives

Overall, the findings in the articles included in this topic suggest that: (A) dysbiosis in the ME and NP microbiotas according to age, temporality with OM onset, and occurrence of LRT disease contribute to OM susceptibility; (B) although transcriptome studies identified novel genes and pathways that are involved in OM susceptibility, many more genes and pathways that are potential targets for novel therapies need to be validated or identified; and (C) animal models remain useful in elucidating mechanisms for OM susceptibility. Future *meta*-omic analyses will help in further understanding the metabolic functions and strain heterogeneity of bacteria in the ME and NP, and will enable detection of microbial factors (e.g., microbial genetic variants, serotypes) that favor resistance to antibiotics or antivirals, biofilm formation, immune evasion, metabolic efficiency, and virulence (Pettigrew et al., 2002; Ecevit et al., 2004; Ehrlich et al., 2005; Shen et al., 2005; Pettigrew et al., 2006; Buchinsky et al., 2007; Hiller et al., 2011; Pettigrew et al., 2011; Thomas et al., 2011; Pettigrew et al., 2012; Lewnard et al., 2016; Hu et al., 2019; Hammond et al., 2020; Harrison et al., 2020). Virulence factors of otopathogens may also identify candidate antigens for novel vaccines (Mottram et al., 2019). The continued study of the confluence of clinical, environmental, genetic, microbiota and immune profiles of patients and animal models with OM will also help identify OM sub-phenotypes that can be useful in personalizing OM treatments.
